# Curcumin inhibits vasculogenic mimicry through the downregulation of erythropoietin-producing hepatocellular carcinoma-A2, phosphoinositide 3-kinase and matrix metalloproteinase-2

**DOI:** 10.3892/ol.2014.2401

**Published:** 2014-07-31

**Authors:** YIMING LIANG, MIN HUANG, JIANWEN LI, XINLIN SUN, XIAODAN JIANG, LIANGPING LI, YIQUAN KE

**Affiliations:** 1 Department of Neurosurgery, Guangzhou Red Cross Hospital, The Fourth Affiliated Hospital of Jinan University, Guangzhou, Guangdong 510200, P.R. China; 2 Department of Neurosurgery, Zhujiang Hospital, Southern Medical University, Guangzhou, Guangdong 510200, P.R. China; 3 Institute of Neurosurgery, Key Laboratory on Brain Function Repair and Regeneration of Guangdong, Southern Medical University, Guangzhou, Guangdong 510200, P.R. China; 4 Neurosurgery Department of Jiangmen Central Hospital, Jiangmen, Guangdong 529030, P.R. China

**Keywords:** curcumin, glioma, matrix metalloproteinase-2, vasculogenic mimicry, erythropoietin-producing hepatocellular carcinoma-A2, phosphoinositide 3-kinase

## Abstract

Glioblastomas (GBMs) are the most common and aggressive malignant primary brain tumors found in humans. In high-grade gliomas, vasculogenic mimicry (VM) is often detected. VM is the formation of *de novo* vascular networks by highly invasive tumor cells, instead of endothelial cells. An understanding of the mechanisms of VM formation will contribute to the targeted therapy of GBMs. In the present study, the efficacy of curcumin (CCM) on VM formation and its mechanisms were investigated. It was found that CCM inhibits the VM formation, proliferation, migration and invasion of human glioma U251 cells in a dose-dependent manner. Furthermore, CCM downregulated the protein and mRNA expression of erythropoietin-producing hepatocellular carcinoma-A2, phosphoinositide 3-kinase and matrix metalloproteinase-2, indicating that CCM may function through these factors for the inhibition of VM formation. These data provide novel insights into the use of CCM to antagonize VM, and may contribute to the angiogenesis-targeted therapy of malignant glioma.

## Introduction

Glioblastomas (GBMs) are tumors that arise from astrocytes, featuring high metastasis, recurrence and mortality rates. Angiogenesis plays an essential role in the growth, proliferation, migration, invasion and metastasis of GBM. Targeting angiogenesis is an important way to treat GBM at present. The angiogenic vascular endothelial growth factor (VEGF)/VEGF receptor (VEGFR) pathway has been considered the most important signaling pathway of GBM angiogenesis (1). However, bevacizumab, a humanized monoclonal antibody directed against VEGF-A, which has recently received regulatory approval for the treatment of recurrent GBM, has limited clinical responses (2,3). Future targeted angiogenesis therapy with effective drugs will provide novel avenues for the treatment of malignant gliomas.

Vasculogenic mimicry (VM) is known as the formation of non-endothelial tumor cell-lined microvascular channels in aggressive tumors, and does not involve endothelial cells. VM is detected predominantly in high-grade gliomas and there is significant association between VM and glioma grade. VM has also been demonstrated to be involved in the proliferation, invasion and metastasis of malignant glioma (4–6). The existence of VM may provide compensation to ensure a blood supply, particularly in less vascularized regions of the tumor. VM may be a novel therapeutic target for anti-angiogenesis in GBM. The molecular mechanisms through which GBM forms VM remain elusive. Previous studies on melanoma have indicated that erythropoietin-producing hepatocellular carcinoma-A2 (EphA2), phosphoinositide 3-kinase (PI3K) and matrix metalloproteinases (MMPs) are the key factors for VM formation (7–11). In melanoma VM, EphA2 is phosphorylated through interactions with its membrane bound ligand, Ephrin-A1. Subsequently, phosphorylated EphA2 activates PI3K, leading to enhanced MMP-14 and -2 expression. MMP-14 and -2 can promote the cleavage of the laminin-5γ2 chain into promigratory γ2′ and γ2× fragments. Fragment release into the tumor microenvironment increases tumor cell migration and invasion, and may ultimately result in VM formation. In malignant glioma VM, it remains unknown whether there is a similar molecular mechanism (10,11).

Curcumin (CCM), a naturally polyphenol derived from the root of the rhizome *Curcuma longa*, possesses anti-inflammatory, antioxidant and anticancer properties. CCM is capable of suppressing carcinogenesis in the forestomach, skin, mammary glands, liver and colon in various animal models *in vivo* (12). Previous studies had indicated that CCM suppresses angiogenesis in malignant tumors (13–15). The anti-angiogenesis mechanism of CCM is involved in various signal pathways, including the downregulated expression of PI3K and MMP-2. As aforementioned, PI3K and MMP-2 have previously been associated with VM formation. We hypothesize that CCM may be able to suppress VM formation. Recently, Chen *et al* reported that CCM inhibited tumor growth and reduced VM in a murine choroidal melanoma model (16). Tumor volume was reduced by CCM and coincidently, the numbers of VM were also significantly decreased. The expression levels of EphA2, PI3K and MMP-2 and -9 were also lower in the treatment group compared with the control group. These observations suggested that CCM had the ability to inhibit the growth of engrafted melanoma VM channels through the regulation of vasculogenic factors that could be associated with the downregulation of the EphA2/PI3K/MMPs signaling pathway. Based on these previous studies, we hypothesized that CCM may affect VM formation in GBM through the regulation of EphA2, PI3K and MMP-2 expression. To test this hypothesis, the present study first analyzed the efficacy of CCM in affecting VM formation in malignant GBM U251 cells, and then examined the expression of EphA2, PI3K and MMP-2 by quantitative PCR and western blot assay following CCM treatment.

## Materials and methods

### Cell culture and experimental groups

The human glioma U251 cell line was obtained from the American Type Culture Collection (Manassas, VA, USA). CCM (C_21_H_20_O_6_; >98% purity) was purchased from Sigma Chemical Co. (St. Louis, MO, USA) and dissolved in dimethyl sulfoxide (DMSO) to create a stock solution, and stored at −20°C until use. The human malignant GBM U251 cell line was maintained in RPMI-1640 cell culture medium supplemented with 10% fetal bovine serum and 100 U/ml penicillin/streptomycin (all Hyclone, GE Healthcare Life Sciences, Logan, UT, USA) in a humidified incubator containing 5% CO_2_ at 37°C. Cultures were passaged every 2–3 days after reaching 80% confluence. Cells in the logarithmic growth phase were used in the experiment.

The cells were divided into the control group (complete culture), the DMSO group (DMSO only, as the negative control group) and the CCM group (treated with CCM at various concentrations, as the treatment group).

### 3-(4,5-dimethylthiazol-2-yl)-5-(3-carboxymethoxyphenyl)-2-(4-sulfophenyl)-2H-tetrazolium (MTS) assay for cell proliferation

The U251 cells were plated at 1×10^4^ cells per well in 96-well culture plates, incubated for 24 h. In accordance with the experimental groups, various concentrations of CCM (2.5, 5, 10, 20, 40 and 80 μM) were added to each well. Subsequent to 12, 24, 48 and 72 h, MTS assays were performed (CellTiter 96^®^ Aqueous One Solution Cell Proliferation Assay, Promega, Madison, WI, USA). MTS was added to each well at a proportion of 1/10 and incubated for 4 h at 37°C. The medium was then removed and 180 μl DMSO was added to each well. The absorbance at 490 nm was measured by a microplate reader (multiscan MK3; Thermo Fisher Scientific, Waltham, MA, USA). The mean cell proliferation was calculated from the absorbance units. All the experiments were carried out in triplicate and each experiment was repeated three times.

### Transwell migration assay

U251 cells at 1×10^5^ per well were seeded into the upper compartment of a Transwell Boyden chamber (BD Biosciences, Franklin Lakes, NJ, USA), with 100 μl serum-free media added into the upper compartment and 600 μl complete media added into the lower compartment. In accordance with the experimental groups, various concentrations of CCM (5, 10, 20 and 40 μM) were added into the upper compartment. Subsequent to incubation for 48 h at 37°C, images of the cells of each group that had migrated to the chamber of the poly carbon membrane were captured and the results quantified.

### Transwell invasion assay

The Matrigel basement membrane matrix (BD Bioscience) was mixed with serum-free medium at a proportion of 1:3, then applied to the upper compartment of the Transwell Boyden chamber, 40 μl per well. Following 30 min of incubation at 37°C, the Matrigel solidified. U251 cells at 1×10^5^ per well were seeded into the upper compartment, 100 μl serum-free media was added into the upper compartment and 600 μl complete media was added into the lower compartment. In accordance with the experimental groups, various concentrations of CCM (5, 10, 20 and 40 μM) were added into the upper compartment. Following incubation for 48 h at 37°C, images of the cells of each group that had migrated to the chamber of the poly carbon membrane were captured and the results quantified.

### In vitro VM tube formation assay

The VM network was established as described previously by Ling *et al* (17). Briefly, the Matrigel basement membrane matrix (BD Biosciences) was thawed at 4°C, mixed quickly with fetal bovine serum at a proportion of 1:3 and then 20 μl was added to each of the 96-well plates. This was allowed to solidify for 2 h at 37°C. The cells were adjusted to 1×10^5^/ml and seeded on the Matrigel-coated plates in a final volume of 200 μl (2×10^4^ cells per well). CCM at various concentrations (5, 10, 20 and 40 μM) was added to each well. The cells were then incubated in a humidified 5% CO_2_ incubator at 37°C. Next, 24 h later, images of each well were captured using phase-contrast microscopy and then quantified.

### Quantitative PCR assay for measurement of the mRNA expression of EphA2, PI3K and MMP-2

To evaluate the mRNA expression of EphA2, PI3K and MMP-2 following CCM treatment, quantitative PCR was performed. Briefly, the U251 cells plated at 1×10^4^ per well in the 96-well culture plates were incubated with various concentrations of CCM (5, 10, 20 and 40 μM) for 48 h. Total RNA from each sample was then isolated with TRIzol reagent (Invitrogen Life Technologies, Carlsbad, CA, USA), according to the manufacturer’s instructions. An optical density assay (RNA 260/280 ratio determined) and agarose electrophoresis analysis showed that the purity and integrity of the RNA was good. Total RNA (1 μg) was used for the reverse transcription process with a Takara PrimeScript™ RT Reagent kit with gDNA Eraser (Takara, Dalian, China), according to the manufacturer’s instruction, and the first-strand cDNA was prepared. The quantitative PCR was performed with Takara SYBR^®^ Premix Ex Taq™ II (Takara) on an ABI PRISM^®^ 7500 Sequence Detection System with a 10-μl total reaction volume consisting of the following: 5 μl 2X SYBR premix EX Taq™, 0.5 μl (10 μM) PCR forward primer, 0.5 μl (10 μM) PCR reverse primer, 0.5 μl cDNA and 3.5 μl dH_2_O. The reaction process was performed at 50°C for 2 min, 95°C for 2 min, 95°C for 15 sec and 60°C for 32 sec, for 40 cycles. The melting curve was analyzed at the temperature range of 60°C–95°C. GAPDH served as an internal reference. The primers were as follows: EphA2 forward, 5′-AAGACCCTGGCTGACTTT-′3 and reverse, 5′-GTTCACCTGGTCCTTGAGT-′3; PI3K forward, 5′-AAAGGCGGCTTGAAAGGT-′3 and reverse, 5′-GACGATCTCCAATTCCCAAA-′3; MMP-2 forward, 5′-CTGGAGATACAATGAGGTGAAG-′3 and reverse, 5′-TCTGAGGGTTGGTGGGATTG-′3; and 18S rRNA forward, 5′-CCTGGATACCGCAGCTAGGA-′3 and reverse, 5′-GCGGCGCAATACGAATGCCCC-′3.

### Western blot assay for measurement of the protein expression of EphA2, PI3K and MMP-2

To evaluate the protein expression of EphA2, PI3K and MMP-2 following CCM treatment, a western blot assay was performed. Briefly, the U251 cells plated at 1×10^4^ per well in 96-well culture plates were incubated with various concentrations of CCM (5, 10, 20 and 40 μM) for 48 h. Protein from each sample was extracted with radioimmunoprecipitation assay cell lysate (CST; Cell Signaling Technology, Inc., Danvers, MA, USA). Protein quantitative analysis was evaluated with a bicinchoninc acid protein assay kit (Pierce Biotechnology, Inc., Rockford, IL, USA). The protein was heated with 5X SDS sample buffer, and 30 μl sample/hole was separated by SDS-PAGE gel electrophoresis, then transferred to polyvinylidene difluoride membranes and washed three times in phosphate-buffered saline with Tween-20 (PBST), for 5 min each time. The membranes were blocked with 5% bovine serum albumin for 1 h at room temperature, and then incubated with the following primary antibodies: Anti-EphA2 (1:500; rabbit polyclonal to Eph receptor A2), anti-PI3K [1:1,000; mouse monoclonal (M253) to PI3K p85-C-termina], anti-MMP-2 [1:400; mouse monoclonal (CA-4001/CA719E3C) to MMP-2) (all Abcam, Cambridge, UK), incubated overnight at 4°C. Subsequent to being thoroughly washed with TBST three times, horseradish peroxidase (HRP)-conjugated secondary antibodies (goat anti-mouse immunoglobulin G-HRP; Wuhan Boster Biological Technology, Ltd., Wuhan, China) were applied, incubated for 2 h at room temperature and washed three times in PBST. Enhanced chemiluminescence light-emitting liquid was then added. GAPDH-HRP (KangChen Bio-tech, Inc., Shanghai, China) was used as an internal reference. The gray scale value of each sample was detected with the fluorescence imaging analyzer (BioPhotometer plus; Eppendorf, Hamburg, Germany).

### Statistical analysis

All experiments were performed three times. The data were recorded as the mean ± standard deviation and evaluated by SPSS 13.0 (SPSS, Inc., Chicago, IL, USA). Differences between groups were analyzed by one way analysis of variance. Repetitive measure analysis of variance was used in analyzing the quality of life at varying time-points. P<0.05 was considered to indicate a statistically significant difference.

## Results

### CCM inhibits the proliferation of U251 cells

**Figure 1:**
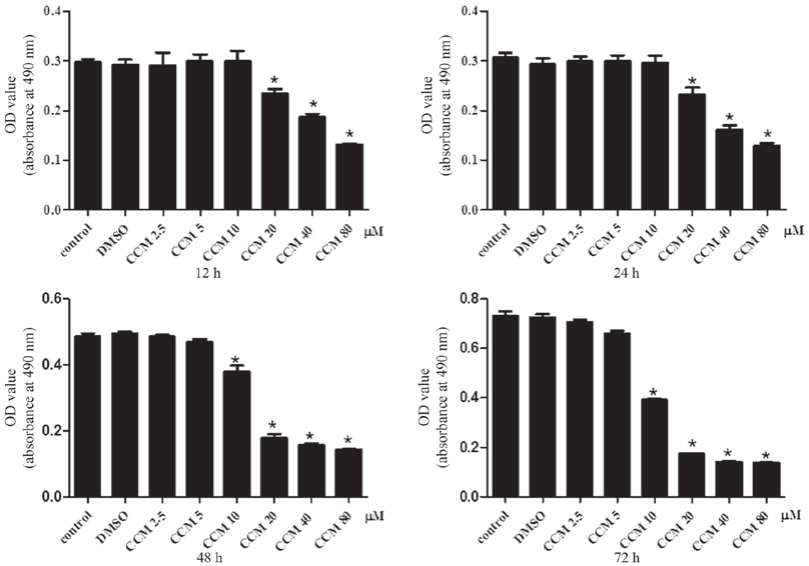
CCM inhibits U251 cell proliferation. The effects of curcumin on cell proliferation were examined by MTS assay. The U251 cells were treated with DMSO or 2.5, 5, 10, 20, 40 and 80 μM CCM for the indicated times. The absorbance at 490 nm was measured. Three independent experiments were performed. Values are expressed as the mean ± standard deviation. ^*^P<0.05 vs. control group. CCM, curcumin; DMSO, dimethyl sulfoxide.

The effect of CCM on U251 cell proliferation was determined using the MTS assay. The U251 cells were treated with CCM at increasing doses (2.5, 5, 10, 20, 40 and 80 μM) and time intervals (12, 24, 48 and 72 h). CCM inhibited the proliferation of the U251 cells in a dose- and time-dependent manner, as shown in Fig. 1.

### CCM inhibits the migration and invasion of U251 cells

**Figure 2:**
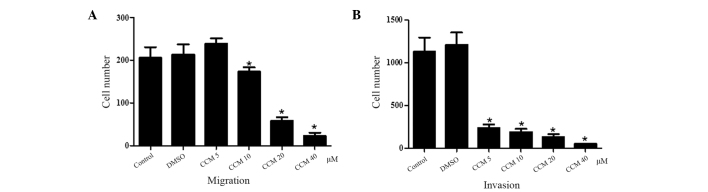
CCM inhibits the (A) migration and (B) invasion of U251 cells in a concentration-dependent manner. The U251 cells were treated with DMSO or 5, 10, 20 and 40 μM CCM for 48 h. Three independent experiments were performed. Values are expressed as the mean ± standard deviation. ^*^P<0.05 vs. control group. CCM, curcumin; DMSO, dimethyl sulfoxide.

The effect of CCM on U251 cell migration and invasion was determined using the Transwell Boyden chamber assay. The migration (Fig. 2A) and invasion (Fig. 2B) of the U251 cells were significantly decreased in a concentration-dependent manner following CCM treatment.

### CCM inhibits VM formation of U251 cells

**Figure 3:**
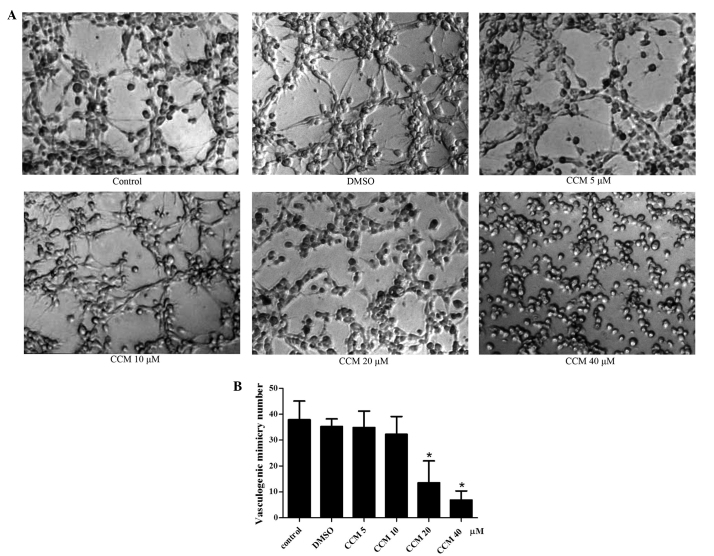
(A) Tubular network structures (VM) were formed by U251 cells on Matrigel (x40 magnification). The CCM inhibited the VM formation of the U251 cells in a dose-dependent manner (x40 magnification). At 20 μM, CCM markedly inhibited the formation of the VM structure by the U251 cells; these levels were significantly different compared with the control group (P<0.05). At 40 μM curcumin, the tubular network structures on the Matrigel were disrupted almost completely (P<0.05). (B) The number of VM structures in every group were quantified. Three independent experiments were performed. Values are expressed as the mean ± standard deviation. ^*^P<0.05 vs. control group. VM, vasculogenic mimicry; CCM, curcumin; DMSO, dimethyl sulfoxide.

In complete medium (control group), the U251 cells gradually grew and adhered, finally connecting with each other and forming tubular network structures on Matrigel, which were judged as the formation of VM. The most typical VM structure were formed at 24 h, The mean number of VM structures per field was 37.88±7.28. Therefore, the 24 h time-point was selected for the observation of the CCM treatment. Next, the U251 cells were treated with curcumin of increasing dose (5, 10, 20 and 40 μM) for 24 h, and the tubular network structures (VM) were observed and quantified. CCM inhibited the VM formation of the U251 cells in a dose-dependent manner, as shown in Fig. 3. When incubated with CCM, the U251 cells lost their ability to form the network structure, the network structures on the Matrigel were disrupted and the number of structures decreased. As the dose of CCM increased, the number of structures gradually decreased. At the doses of 5 and 10 μM, CCM inhibited the VM formation slightly, but with no significant difference compared with the control group. At the dose of 20 μM, CCM markedly inhibited the formation of the VM structure by the U251 cells; this was significantly different compared with control group (P<0.05). At 40 μM CCM, the network structures on the Matrigel were disrupt almost completely (P<0.05).

### CCM suppresses the mRNA expression of EphA2, PI3K and MMP-2 in U251 cells

**Figure 4:**
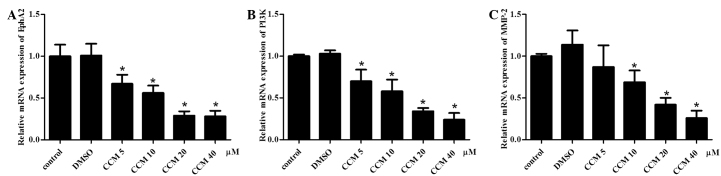
mRNA expression levels of EphA2, PI3K and MMP-2 following CCM treatment, examined by quantitative PCR. GAPDH served as an internal reference. The CCM suppressed the mRNA expression level of (A) EphA2, (B) PI3K and (C) MMP-2 in a dose-dependent manner. Three independent experiments were performed. Values are expressed as the mean ± standard deviation. ^*^P<0.05 vs. control group. CCM, curcumin; DMSO, dimethyl sulfoxide.

The mRNA expression of EphA2, PI3K and MMP-2 following CCM treatment were detected with quantitative PCR. GAPDH served as an internal reference. The mRNA expression of EphA2, PI3K and MMP-2 in the CCM groups (5, 10, 20 and 40 μM) was downregulated. The CCM suppressed the mRNA expression of EphA2, PI3K and MMP-2 in the U251 cells in a dose-dependent manner, as shown in Fig. 4. As the dose of CCM increased, the mRNA expression of EphA2, PI3K and MMP-2 decreased. At the doses of 10, 20 and 40 μM, CCM markedly suppressed the mRNA expression of EphA2, PI3K and MMP-2, respectively; these levels were significantly different compared with the control group (P<0.05).

### CCM decreases the protein level of EphA2, PI3K and MMP-2 in U251 cells

**Figure 5:**
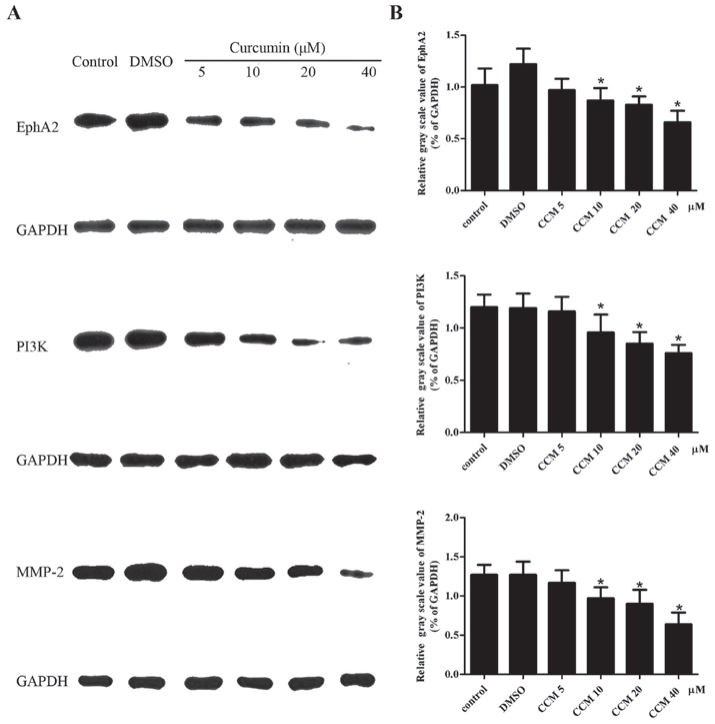
(A) The protein levels of EphA2, PI3K and MMP-2 in the U251 cells were detected by western blot assays. GAPDH served as an internal reference. (B) CCM suppressed the expression of the EphA2, PI3K and MMP-2 proteins in a dose-dependent manner. Three independent experiments were performed. Values are expressed as the mean ± standard deviation. ^*^P<0.05 vs. control group.

The protein levels of EphA2, PI3K and MMP-2 in the U251 cells were detected by western blot assays. GAPDH served as an internal reference. The protein levels of EphA2, PI3K and MMP-2 in the CCM groups (5, 10, 20 and 40 μM) were decreased. CCM suppressed the expression of the EphA2, PI3K and MMP-2 proteins in a dose-dependent manner, as shown in Fig. 5. At the doses of 10, 20 and 40 μM, CCM markedly decreased the protein level of EphA2, PI3K and MMP-2, respectively; these levels were significantly different compared with control group (P<0.05).

## Discussion

CCM has become a focus of attention due to its inhibitory role in GBM. CCM has been found to suppress the initiation, proliferation, migration and metastasis of glioma cells *in vitro* and *in vivo*. The effective mechanisms of CCM include cell cycle arrest, the induction of apoptosis, the suppression of oncogenes and the enhancement of tumor suppressor genes (18–22). Molecular targets, including the PI3K/Akt/mTOR, protein kinase C, Ras/mitogen-activated protein kinase, Wnt and intrinsic or extrinsic apoptosis pathways have been studied (23). In the present study, CCM suppressed the proliferation of the U251 cells in a dose- and time-dependent manner, as determined by MTS assay. The Transwell Boyden chamber assays showed that the migration and invasion of the U251 cells were significantly impaired in a concentration-dependent manner following CCM treatment. Moreover, downregulation of the EphA2, PI3K and MMP-2 expression was associated with the inhibition of GBM by CCM.

Inhibition of angiogenesis is one of the important mechanisms of action for CCM against GBM. At present, studies on the effect of CCM against GBM angiogenesis mainly focus on endothelium-dependent angiogenesis (24). The mechanism of CCM against GBM angiogenesis involves a number of signaling pathways and effective molecules. CCM has been shown to inhibit the expression of VEGF (25). CCM also inhibits the constitutive activation of the PI3K/Akt pathway, which is upregulated in glioma cell lines (22). Abnormal MMP expression is significant in malignant glioma invasion into the surrounding normal brain tissues. It has previously been shown that CCM has broad-spectrum inhibitory activity against MMP gene expression in human astroglioma cells. CCM has also been demonstrated to inhibit the expression of MMP-2 and -9 in human astroglioma cells (26,27). Since there is cross-talk between the aforementioned pathway-related molecules and the VM formation-related molecules, the question of whether CCM can inhibit the formation of VM in GBM has arisen. If CCM can inhibit endothelium-dependent angiogenesis and the formation of VM in GBM, it can become an ideal anti-angiogenesis drug for treating GBM.

In the current study, it was reported that CCM inhibited the VM formation of the U251 cells in a dose-dependent manner. When incubated with CCM, the U251 cells lost their ability to form the network structure, the network structures on the Matrigel were disrupted and the number of structures decreased. As the dose of CCM increased, the inhibition of VM formation also increased. At the doses of 10, 20 and 40 μM, CCM markedly inhibited the formation of the VM structure by the U251 cells; these levels were significantly different compared with the control group (P<0.05).

Subsequently, the present study explored the mechanism of CCM inhibition on the VM formation of the U251 cells. We hypothesized that EphA2, PI3K and MMP-2 may be the critical factors in VM formation in GBM. Therefore, EphA2, PI3K and MMP-2 were examined by quantitative PCR and western blot assays following CCM treatment in the U251 cells. It was found that the mRNA expression levels of EphA2, PI3K and MMP-2 in the CCM groups (5, 10, 20 and 40 μM) were downregulated, and that the protein expression levels were decreased. In the U251 cells, CCM suppressed the expression of EphA2, PI3K and MMP-2 mRNA and protein in a dose-dependent manner.

A growing body of evidence has suggested that EphA2, PI3K and MMP-2 play significant roles in the formation of VM in GBM. Firstly, EphA2, PI3K and MMP-2 are highly expressed in GBM, and their expression levels are positively correlated with the pathological grade, proliferation and invasion of GBM. Immunohistochemical studies have revealed that the presence of VM is associated with the expression of MMP-2, MMP-14, EphA2 and laminin 5γ2 in medulloblastoma (4). The PI3K/AKT signaling pathway regulates glioma cell proliferation and invasion, while pharmacological inhibitors of PI3K/Akt signaling (LY294002 and A443654) reduce the motility of GBM cells and diminish the level of MMP-2 activity and MMP-2 expression (28,29). Penta-acetyl geniposide has been shown to exhibit an inhibitory effect on the abilities of adhesion and motility in C6 glioma cells, decreased the expression of MMP-2 and inhibited the expression of PI3K protein (30).

Secondly, EphA2, PI3K and MMP-2 may take part in the formation of VM in GBM. Wu *et al* (31) found that ectopic expression of miR-26b inhibited the proliferation, migration and invasion of human glioma cells. In addition, miR-26b inhibited the VM processes accompanied with the downregulation of EphA2 proteins, which revealed that EphA2 was correlated with the capability of VM formation in gliomas. Ling *et al* (17) blocked the expression of MT1-MMP, a cell surface activator of MMP-2-pro in glioma, by small interfering (si)RNA transient transfection in U251MG, and found that the VM formation was significantly impaired and that the activity of MMP-2 in the MT1-MMP siRNA group was significantly decreased. However, Ling *et al* (17) also showed that EphA2 may not be involved in TGFβ-induced VM formation. In the current study, it was found that the expression levels of EphA2, PI3K and MMP-2 were inhibited by CCM treatment, which was accompanied with a decrease in VM formation in the U251 cells. This result was similar to the results of studies on melanoma, and indicated that the expression of EphA2, PI3K and MMP-2 may be responsible for the capability of VM formation in gliomas (16). Future studies should confirm these observations *in vivo*.

In conclusion, the present study demonstrated for the first time that CCM inhibits the VM formation of U251 cells and downregulates the expression of EphA2, PI3K and MMP-2. The results provide new evidence for the use of CCM in the treatment of malignant glioma, and may contribute to the angiogenesis-targeted therapy for this disease.
